# Potential drug targets for asthma identified through mendelian randomization analysis

**DOI:** 10.1186/s12931-024-03086-5

**Published:** 2025-01-13

**Authors:** Xingxuan Chen, Yu Shang, Danting Shen, Si Shi, Zhe Wen, Lijuan Li, Hong Chen

**Affiliations:** 1 Department of Pulmonary and Critical Care Medicine, The Second Affiliated Hospital of Harbin Medical University, Harbin, 150086 China; 2 Department of Respiratory Medicine, The Second Hospital of Heilongjiang Province, Harbin, 150001 China; 3 Department of Pulmonary and Critical Care Medicine, National Clinical Research Center of Respiratory Diseases, Friendship Hospital, 100029 Beijing, China

**Keywords:** Asthma, Drug targets, Mendelian randomization, Therapeutic targets, Cerebrospinal fluid proteins, Plasma proteins

## Abstract

**Background:**

The emergence of new molecular targeted drugs marks a breakthrough in asthma treatment, particularly for severe cases. Yet, options for moderate-to-severe asthma treatment remain limited, highlighting the urgent need for novel therapeutic drug targets. In this study, we aimed to identify new treatment targets for asthma using the Mendelian randomization method and large-scale genome-wide association data (GWAS).

**Methods:**

We utilized GWAS data from the UK Biobank (comprising 56,167 patients and 352,255 control subjects) and the FinnGen cohort (including 23,834 patients and 228,085 control subjects). Genetic instruments for 734 plasma proteins and 154 cerebrospinal fluid proteins were derived from recently published GWAS. Bidirectional Mendelian randomization analysis, Steiger filtering, colocalization, and phenotype scanning were employed for reverse causal inference detection, further substantiating the Mendelian randomization results. A protein-protein interaction network was also constructed to reveal potential associations between proteins and asthma medications.

**Results:**

Under Bonferroni significance conditions, Mendelian randomization analysis revealed causal relationships between seven proteins and asthma. In plasma, we observed that an increase of one standard deviation in IL1R1[1.30 (95% CI 1.20–1.42)], IL7R[1.07 (95% CI 1.04–1.11)], ECM1[1.03 (95% CI 1.02–1.05)], and CD200R1[1.18 (95% CI 1.09–1.27)] were associated with an increased risk of asthma, while an increase in ADAM19 [0.87 (95% CI 0.82–0.92)] was found to be protective. In the brain, each 10-fold increase in IL-6 sRa [1.29 (95% CI 1.15–1.45)] was associated with an increased risk of asthma, while an increase in Layilin [0.61 (95% CI 0.51–0.73)] was found to be protective. None of the seven proteins exhibited a reverse causal relationship. Colocalization analysis indicated that ECM1 (coloc.abf-PPH4 = 0.953), IL-6 sRa (coloc.abf-PPH4 = 0.966), and layilin (coloc.abf-PPH4 = 0.975) shared the same genetic variation as in asthma.

**Conclusion:**

A causal relationship exists between genetically determined protein levels of IL1R1, IL7R, ECM1, CD200R1, ADAM19, IL-6 sRa, and Layilin (LAYN) and asthma. Moreover, the identified proteins may serve as attractive drug targets for asthma, especially ECM1 and Layilin (LAYN). However, further research is required to comprehensively understand the roles of these proteins in the occurrence and progression of asthma.

**Supplementary Information:**

The online version contains supplementary material available at 10.1186/s12931-024-03086-5.

## Background

Asthma is a chronic inflammatory respiratory disease characterised by airway inflammation, hypersensitivity, and hyperresponsiveness, leading to symptoms such as breathing difficulties, coughing, chest tightness, and wheezing [1, 2]. Asthma exhibits significant heterogeneity in clinical symptoms, severity, and treatment outcomes [3]. With an in-depth understanding of the pathogenesis of asthma and continuous advancements in drug development technologies, an increasing number of novel drug targets have emerged and been applied in clinical treatment [4, 5]. These new drugs act on key processes in the pathophysiology of asthma through various mechanisms, including anti-inflammatory effects, airway dilation, and modulation of immune responses [6], bringing new hope and opportunities for asthma treatment. However, there are significant variations in the response to medication among different patients in clinical settings, posing a substantial therapeutic challenge for both healthcare teams and patients. In addition to daily management strategies, there is a growing need for novel therapeutic targets to address this challenge.

Although large-scale randomised controlled trials (RCTs) are effective for evaluating drug treatment strategies, but they are both time-consuming and expensive [7]. Genome-wide Association Studies (GWAS) have identified numerous genetic loci associated with disease risk [8, 9], providing evidence for determining the molecular pathways involved in drug interventions for diseases. Moreover, evidence suggests that drug targets with human genetic support are more than twice as likely to be approved as those without [10, 11]. Therefore, incorporating genetics into drug development is feasible.

**Fig. 1:**
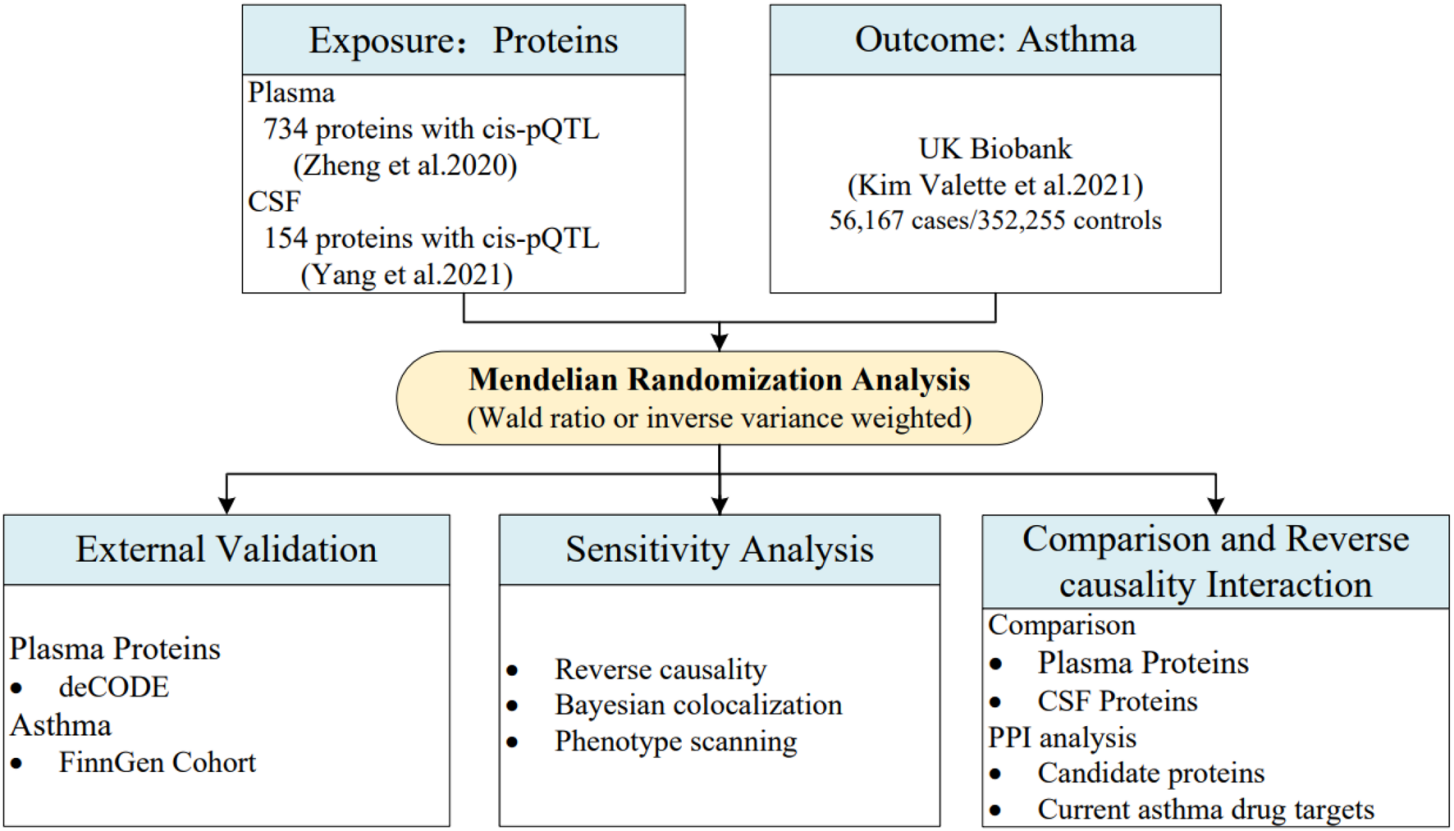
Study design for the identification of plasma and brain proteins causally associated with asthma

Mendelian randomization (MR) is a statistical analysis in genetics that can predict the efficacy of drugs by mimicking randomised controlled trials, and has been widely used in drug target development and drug repurposing [12–14]. Leveraging genome-wide association study (GWAS) data, MR utilizes single nucleotide polymorphisms (SNPs) closely associated with exposure as genetic tools to infer whether the association between exposure and outcome is causal. GWAS of plasma protein levels have identified genetic variations associated with proteins, commonly known as protein quantitative trait loci (pQTLs), which can guide the search for pathogenic genes and disease pathways, providing opportunities to use Mendelian randomization to explore drug targets [15, 16]. Combining human genetics with high-throughput, population-scale proteomics helps to explore the relationship between the human genome and diseases. So far, few Mendelian randomization (MR) studies have explored drug targets for asthma using Protein Quantity Trait Loci (pQTL). In this study, we used the Mendelian randomization method and large-scale genome-wide association research data to identify new therapeutic targets for asthma. Figure 1 describes the design of this study.

## Methods

### Data

Cerebrospinal fluid pQTL (CSF pQTL) data were obtained from a study reporting 274 pQTLs for 184 CSF proteins [17]. The study included individuals with AD and cognitively normal individuals of European ancestry. Plasma pQTL data were retrieved from another study [18] that consolidated 3606 pQTLs for 2656 proteins using five previously published GWAS datasets [19–23]. Plasma pQTL data are almost from European ancestry.Three MR assumptions influence the genetic instrumental variables used for MR analysis, so we only included pQTLs that met the following criteria: (1) showing genome-wide significant associations (*P* < 5 × 10^−8^); (2) located outside the major histocompatibility complex (MHC) region of humans (chr6, 26–34 Mb); (3) exhibiting independent associations [linkage disequilibrium (LD) r^2^ < 0.001]; (4) being cis-acting pQTL; and (5) having an F-statistic > 10 [F = (beta/se)^2^]. Finally, 154 cis-acting pQTLs for 154 proteins and 738 cis-acting SNPs for 734 proteins were included from the CSF and plasma proteins, respectively (Additional file 1:Table S1). Data checks were conducted on the original studies to ensure reliability. Additionally, the corresponding plasma pQTL data (4,907 plasma proteins measured in 35,559 Icelanders) were extracted from a study by Ferkingstad et al. [24] for external validation.

Asthma data were derived from the GWAS data of the UK Biobank, comprising 56,167 patients and 352,255 control subjects of European ancestry [25]. Summary data for the GWAS analysis were sourced from the IEU Open GWAS project and can be downloaded from https://gwas.mrcieu.ac.uk/. Asthma data for external validation were obtained from the FinnGen cohort [26] (23,834 patients and 228,085 control subjects of European ancestry; https://www.finngen.fi/en).

### MR Analysis

The R package TwoSampleMR (version 4.3.1) (https://github.com/MRCIEU/TwoSampleMR) was used for MR analysis. Exposure and outcome data were imported and harmonised using built-in functions within the R package (harmonise_data). If only one pQTL was available for a given protein, the Wald ratio was used to compute the MR estimate for each SNP. When two or more instrumental variables were available, the inverse variance weighted (IVW) method was applied, followed by a heterogeneity analysis [27]. Odds ratios (OR) for increased asthma risk were presented as the increase in the standard deviations (SD) of plasma protein levels and a 10-fold increase in CSF protein levels.

In the primary analysis, considering the false positives caused by multiple testing, Bonferroni correction was applied to determine the significance threshold after multiple testing, with a P-value < 5.63 × 10^−5^ (*P* = 0.05/888) defined as significant [28]. For external validation, MR analysis was performed only on the initially identified proteins, with the P-value threshold set at 0.05. The preliminary findings were validated by extracting the same SNPs used in the primary analysis from the database as genetic instruments and by using the most significant SNP for that protein across the genome in the same database as a genetic instrument.

### Colocalization analysis

Colocalization analysis was used to identify whether the two phenotypes were driven by the same causal variant in a particular region, thereby strengthening the evidence of association between the two phenotypes. colocalization analysis for asthma risk is conducted using the R package “coloc” (https://github.com/chr1swallace/coloc). In a given region, the posterior probabilities obtained from the Colocalization analysis correspond to one of the following five hypotheses: PPH0, SNPs unrelated to either trait; PPH1, SNPs related to protein expression but not asthma risk; PPH2, SNPs related to asthma risk but not protein expression; PPH3, SNPs related to both asthma risk and protein expression but driven by different SNPs; and PPH4, SNPs related to both asthma risk and protein expression and driven by a common SNP. The significance threshold for colocalization was set at PPH4 > 0.80, where proteins colocalising with asthma risk can be considered potential drug targets [29].

### Reverse causality detection

Using asthma data from the primary analysis as exposure and the initially identified proteins as outcome(Additional file 1: Table S2), a bidirectional MR analysis was conducted to detect potential reverse causal relationships. The inverse variance-weighted method (IVW) [30], weighted mode (WM), weighted median method (WME), simple mode (SM), and MR-Egger regression methods were employed for effect estimation. Statistical significance was set at *P* < 0.05^31^.

### Phenome-wide scan

To further assess the pleiotropic effects of potential drug targets, we used the selected pQTLs as key terms to search the literature for associations with other traits. The PhenoScanner (http://www.phenoscanner.medschl.cam.ac.uk/) was used to investigate whether SNPs were significantly associated with other traits and any known asthma risk factors, including metabolic traits, proteins, or clinical characteristics.

### Plasma and cerebrospinal fluid protein comparative analysis

We hypothesised that due to the blood-brain barrier, there is a minimal correlation between pQTLs identified in the plasma and cerebrospinal fluid. Therefore, we investigated the correlation between pQTLs identified in the cerebrospinal fluid and the effect estimates of plasma proteins using MR analysis through Spearman correlation analysis. Different P-value thresholds were set to explore whether the correlation changed with increasing significance levels.

### Constructing protein-protein interaction networks

Protein-protein interaction (PPI) networks consist of proteins that interact with each other, contributing to various biological processes such as signal transduction, gene expression regulation, energy and substance metabolism, and cell cycle control. By evaluating and analysing PPI networks, researchers can gain insights into how proteins interact within cells.

In this study, we utilised the Search Tool for the Retrieval of Interacting Genes (STRING) database (https://string-db.org/) with a confidence score of 0.4^32^ to investigate the interactions among significant proteins. The PPI results were further visualised using Cytoscape (V3.9.1) [33]. Additionally, we summarised the existing therapeutic targets for asthma on the market and explored the corresponding drug targets based on the DrugBank database (https://www.drugbank.ca) associated with these asthma-related genes to predict potential therapeutic drugs [34].

## Results

### Screening for asthma-causing proteins in the proteome

**Fig. 2:**
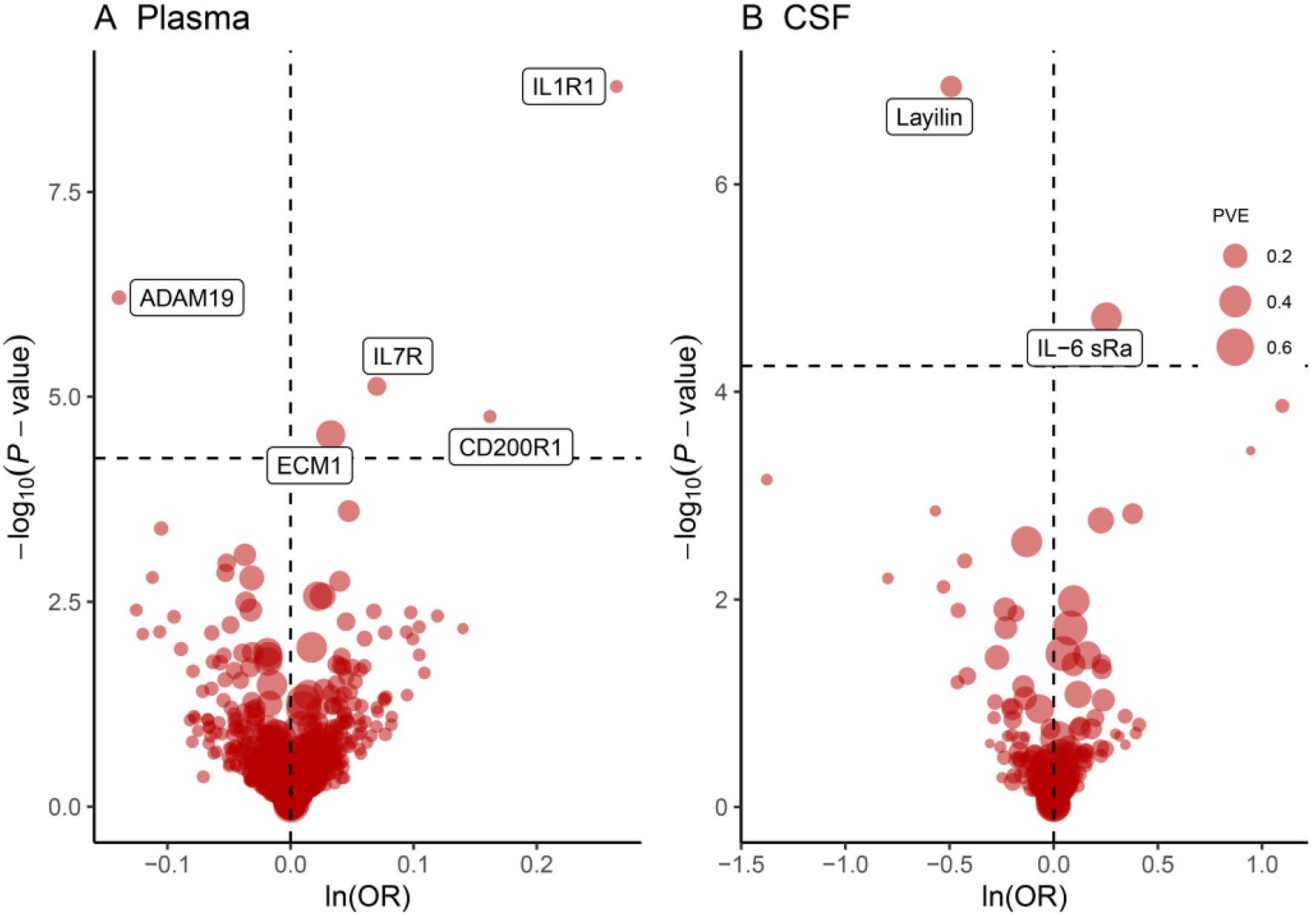
MR results for plasma and CSF proteins and the risk of astnma.Volcano plots of the MR results for **A** 734 plasma and **B** 154 CSF proteins on the risk of asthma. **A** and **B** show MR analysis with Wald ratio or inverse variance weighted method on plasma and CSF proteins on the risk of asthma, respectively. OR for increased risk of asthma were expressed as per SD increase in plasma protein levels and per 10-fold increase in CSF protein levels. Dashed horizontal black line corresponded to *P* = 5.63 × 10^−5^ (0.05/888). ln = natural logarithm; PVE = proportion of variance explained

We conducted an MR analysis of 154 proteins in the cerebrospinal fluid and 734 proteins in the plasma. Under Bonferroni significance (*P* < 5.63 × 10^−^5), seven proteins were found to be associated with asthma risk (Table 1; Fig. 2A and B), including five plasma proteins: interleukin 1 receptor type 1 (IL1-R1), interleukin 7 receptor (IL-7R), extracellular matrix protein 1 (ECM1), CD200 receptor 1 (CD200R1), ADAM metallopeptidase domain 19 (ADAM19), and two cerebrospinal fluid proteins: IL-6 sRa (interleukin 6 receptor, IL6R) and Layilin (LAYN).

**Table 1:** MR results for plasma and CSF proteins significantly associated with asthma after

Tissue	Protein	UniProt ID	SNPa	Effect allele	OR (95% CI)b	*P* value	PVE	F statistics	Author
Plasma	IL1R1	P14778	rs7588201	A	1.30 (1.20, 1.42)	1.63E-09	1.21%	39.3	Emilsson
Plasma	IL7R	P16871	rs11957503	G	1.07 (1.04, 1.11)	7.48E-06	8.69%	94.74	Suhre
Plasma	ECM1	Q16610;A0A140VJI7	rs13294	A	1.03 (1.02, 1.05)	2.92E-05	36.97%	584.28	Suhre
Plasma	CD200R1	Q8TD46	rs6791672	A	1.18 (1.09, 1.27)	1.74E-05	1.45%	48.72	Sun
Plasma	ADAM19	Q9H013	rs7728609	C	0.87 (0.82, 0.92)	6.17E-07	2.72%	89.52	Emilsson
CSF	IL-6 sRa	P08887	rs4129267	T	1.29 (1.15, 1.45)	1.93E-05	36.34%	476.66	Yang
CSF	Layilin	Q6UX15	rs674230	G	0.61 (0.51, 0.73)	1.14E-07	14.18%	137.92	Yang

Bonferroni correction

PVE = proportion of variance explained

^a^All SNPs used were cis-acting

^b^Odds ratios for increased risk of asthma were expressed as per SD increase in plasma protein levels and per 10-fold increase in CSF protein levels

Specifically, an increase in IL1-R1 (OR = 1.30; 95% CI 1.20–1.42, *P* = 1.63 × 10^−9^), IL-7R (OR = 1.07; 95% CI 1.04–1.11, *P* = 7.48 × 10^−6^), ECM1 (OR = 1.03; 95% CI 1.02–1.05, *P* = 2.92 × 10^−5^), CD200R1 (OR = 1.18; 95% CI 1.09–1.27, *P* = 1.74 × 10^−5^), and IL-6 sRa (OR = 1.29; 95% CI 1.15–1.45; *P* = 1.93 × 10^−5^) increased the risk of asthma. Conversely, an increase in ADAM19 (OR = 0.87; 95% CI 0.82–0.92; *P* = 6.17 × 10^−7^) and Layilin (OR = 0.61; 95% CI 0.51–0.73; *P* = 1.14 × 10^−7^) decreased the risk of asthma. The heterogeneity tests revealed no significant heterogeneity or outliers(Additional file 1:Table S3).

### Sensitivity analysis

Due to the pleiotropic effects of instrumental variables (IVs) on MR, we conducted several sensitivity analyses. First, reverse MR analysis on the seven identified proteins and asthma did not reveal any causal effects of asthma on the levels of the seven identified proteins, and further directionality was ensured through Steiger filtering (Table 2 and Supplementary Fig. 1). Second, using the coloc package in R, we conducted colocalization analysis on genes within ± 1 Mb regions (upstream or downstream) of the seven identified pQTLs to further identify causative genetic variants associated with asthma, using PPH4 > 0.80 as a significant threshold. The results indicated that the plasma protein ECM1 shared causative variation with asthma (PP.H4 = 0.95), and the cerebrospinal fluid proteins IL-6 sRa (PP.H4 = 0.97) and Layilin (PP.H4 = 0.98) shared causative variations with asthma Table 2, Supplementary Fig. 2 and Additional file 1: Table S7. Therefore, three potential druggable proteins were identified from the colocalization analysis, providing evidence of shared genetic effects between pQTLs and asthma risk.

**Table 2:** Summary of reverse causality detection, bayesian co-localization analysis and phenotype scanning on seven potential causal proteins

Tissue	Protein	UniProt ID	SNP	Bidirectional MR (MR-IVW)^a^	Steiger filtering	coloc.abf. PPH4	Previously reported associations
Plasma	IL1R1	P14778	rs7588201	1.038 (0.985–1.093)	Ture 1.201 × 10^−8^	0.016	Eczema ^b^ Allergic disease ^c^
Plasma	IL7R	P16871	rs11957503	0.973 (0.941–1.007)	Ture 8.946 × 10^−21^	0.022	White blood cell ^b^
Plasma	ECM1	Q16610;	rs13294	0.980 (0.944–1.017)	Ture 1.536 × 10^−107^	0.953	Monocyte ^c^ Blood platelet ^c^ Atopic dermatitis ^b^
Plasma	CD200R1	Q8TD46	rs6791672	0.986 (0.953–1.021)	Ture 5.767 × 10^−11^	0.181	Eczema ^c^ Allergic rhinitis ^b^
Plasma	ADAM19	Q9H013	rs7728609	0.980 (0.948–1.013)	Ture 3.876 × 10^−19^	0.0004	1 s forced expiratory volume ^c^ Peak expiratory flow ^c^ Age-related macular degeneration. ^b^
CSF	IL-6 sRa	P08887	rs4129267	1.016 (0.953–1.021)	Ture 2.281 × 10^−88^	0.966	Red blood cell Distribution width ^c^ Coronary artery disease. ^b^
CSF	Layilin	Q6UX15	rs674230	0.954 (0.912–0.998)	Ture 4.868 × 10^−29^	0.975	Allergic disease ^c^

MR-IVW = Mendelian randomization with inverse variance weighted method; PP = posterior probability;

^a^ Odds ratios per SD increase in plasma protein levels and per 10-fold increase in CSF protein levels as asthma risk increased

^b^ SNP associated with traits directly

^c^ SNP associated with traits mediated by its proxy

Third, by reviewing the literature and using PhenoScanner, we explored the potential confounding factors for the identified proteins. The results showed that IL1-R1 is associated with leukocytes and allergic diseases in humans, such as neutrophils and eosinophils; IL-7R is related to leukocytes in humans, including neutrophils, eosinophils, basophils, and lymphocytes; ECM1 is associated with monocytes, platelets, and atopic dermatitis; CD200R1 is related to eosinophils, neutrophils, basophils, hay fever, allergic rhinitis, or eczema in humans; ADAM19 is associated with forced expiratory volume in one second, peak expiratory flow rate, age-related macular degeneration; IL-6 sRa is related to leukocytes, haemoglobin concentration, mean corpuscular haemoglobin, monocytes, red cell distribution width, coronary artery disease in humans; Layilin is associated with allergic diseases. Asthma is an allergic disease, and we found that IL1R1 and Layilin are both associated with allergic diseases, whereas ECM1 is related to atopic dermatitis. Atopic dermatitis and asthma have been previously reported, indicating that these diseases may share a common aetiology. Furthermore, no significant confounding factors were identified(Table 2 and Additional file 1:Table S4).

### Comparison of proteins in plasma and cerebrospinal fluid

At the protein level, a non-significant positive correlation (Spearman correlation coefficient = 0.044) was observed between the MR results of cerebrospinal fluid and plasma. Additionally, when using different P-value threshold restrictions to limit the number of proteins included in the analysis, the positive correlation persisted and remained non-significant (Supplementary Fig. 3).

Next, the three potential drug target proteins identified through colocalization screening were loaded into the STRING database (https://cn.string-db.org/) for network construction. The resulting file was imported into Cytoscape for visualisation of protein-protein interaction (PPI) networks, which displayed interactions between the three drug-target proteins and other proteins (Supplementary Figure S4).

**Fig. 3:**
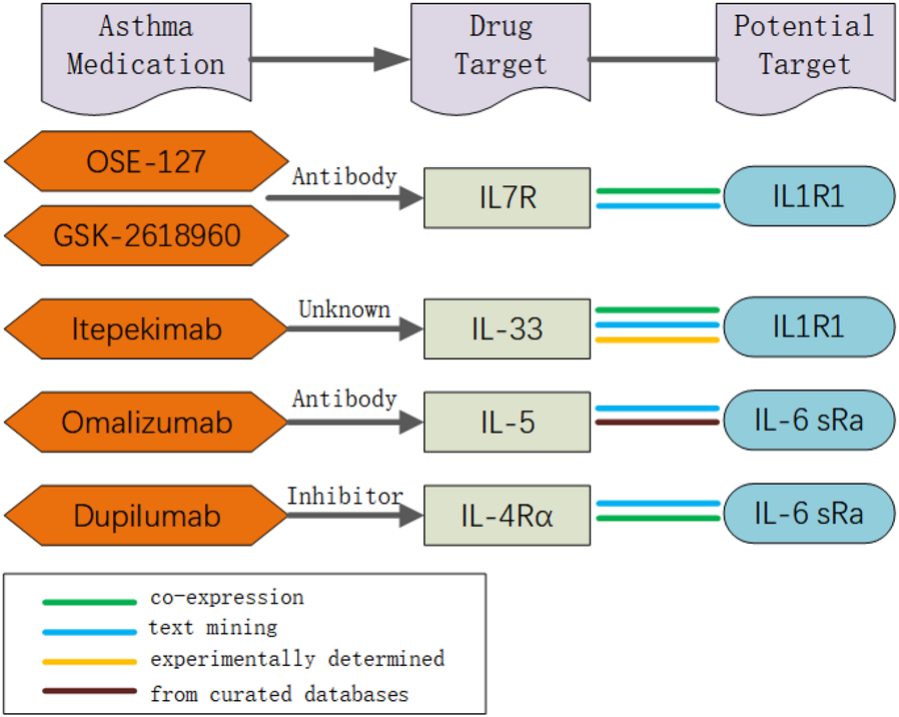
Interaction between current asthma medications targets and identified potential drug targets

Furthermore, we constructed a PPI network with the additional four identified proteins and three potential drug target proteins, which revealed interactions between different proteins (Supplementary Figure S5). Notably, we observed a strong and reliable interaction between IL1-R1 and IL-7R. IL1-R1 is associated with anti-IL7R monoclonal antibodies (IL-7R), and IL-7R is the target of OSE-127 and GSK-2,618,960 (Fig. 3 and Additional file 1:Table S5).

Association between potential drug targets and current asthma medications. We constructed a protein-protein interaction (PPI) network by associating three potential drug targets with four asthma drug targets (IgE, IL-5, IL-4, and TSLP). Seven proteins were loaded into the STRING database for network creation and the resulting file was imported into Cytoscape for PPI network visualisation(Supplementary Figure S6). We found that IL-6 sRa (IL-6R) is associated with IL-5 and IL-4. The latter are targets of the following asthma drugs: anti-IL-5/IL-5R monoclonal antibodies (Mepolizumab, Benralizumab, Reslizumab), and anti-IL-4Rα monoclonal antibody (Dupilumab) (Fig. 3). We also searched the DrugBank database for drugs targeting the identified potential pathogenic proteins(Additional file 1:Table S6).

### External validation of asthma

**Fig. 4:**
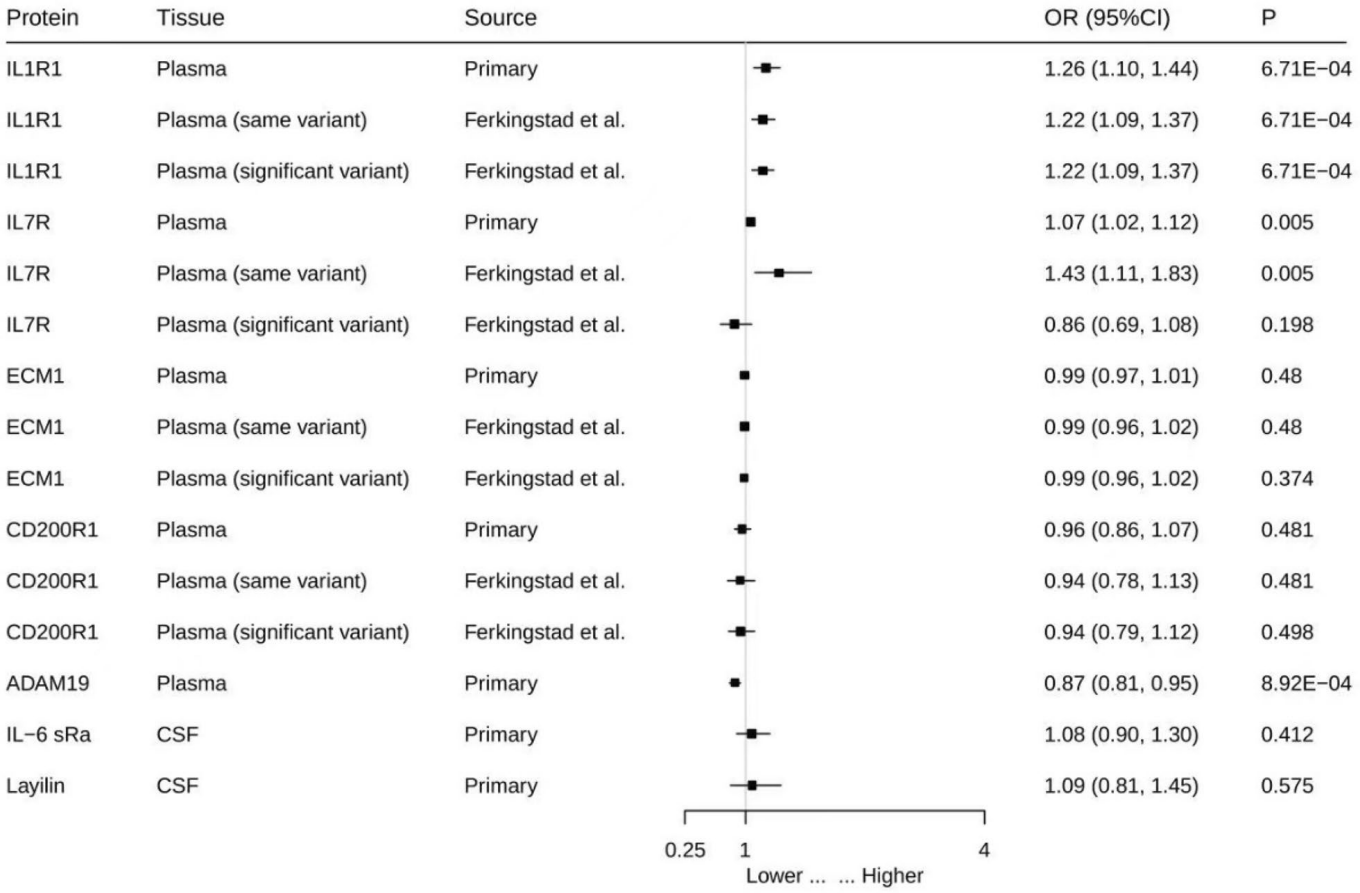
External validation of the causal relationship between seven potential causal proteins and asthma MR analysis on the causal relationship of six potential causal proteins on asthma using data from the FinnGen cohort. OR for increased risk of MS were expressed as per SD increase in plasma protein levels and per 10-fold increase in CSF protein levels

Based on the primary analysis of pQTL data, we searched for the same variants in different datasets for external validation as well as significant variants unique to different datasets to enhance the scientific rigor of Mendelian randomization studies on drug target identification. Plasma pQTL data for exposure were extracted from the study by Ferkingstad et al., whereas asthma data for outcomes were obtained from the FinnGen database. The results revealed that IL1-R1 was also found to be associated with asthma in different databases, with an increased risk of asthma associated with elevated IL1-R1 levels (OR = 1.22; 95% CI 1.09–1.37, *p* = 6.71 × 10^−4^). ADAM19 and IL-7R were also associated with asthma outcomes in the FinnGen database when the same variants identified in the primary analysis were used as exposure factors(Fig. 4 and Additional file 1:Table S8).

## Discussion

Research has demonstrated the efficacy of targeted drug therapy in improving asthma control; however, significant variations in drug responses exist among patients, underscoring the pressing need for more effective targeted therapies. Therefore, the quest for viable for asthma treatment targets is paramount. To date, Li et al. [35] have identified asthma genes through eQTL analysis of bronchial epithelial cells and bronchoalveolar lavage fluid; Zaid et al. [36] and Nieuwenhuis et al. [37] have identified a series of asthma drug targets based on GWAS and eQTL analysis; Wang et al. [38] have identified a series of asthma drug targets based on GWAS and pQTL analysis. In our study, using a larger cerebrospinal fluid protein database and plasma protein identification, we identified more comprehensive asthma-targeted protein sites. We identified seven proteins associated with asthma risk, three of which may serve as new asthma treatment targets. Furthermore, using protein-protein interaction networks and DrugBank, we identified drugs that may have therapeutic potential for patients with asthma. Our study complements previous related research by identifying seven proteins with a causal relationship with asthma risk. Through colocalization analysis of the seven initially identified proteins, three proteins were identified as potential drug targets for asthma, including ECM1, IL-6 sRa, and layilin. Additionally, using the same analysis method in the FinnGen database, we found that IL1-R1, ADAM19, and IL7R were associated with asthma risk, further demonstrating the stability of the results obtained in this study.

Asthma is characterised by airway hyper-responsiveness and excessive bronchoconstriction [39]. Brain-derived neurotrophic factors actively recruit eosinophils, stimulate their degranulation, and release major basic proteins, thereby enhancing parasympathetic nerve-mediated bronchoconstriction [40]. Simultaneously, neurogenic inflammation can trigger asthma attacks by releasing neuropeptides via local axon reflexes [41]. This further prompted us to conduct an MR analysis of cerebrospinal fluid proteins associated with asthma risk to explore the causal relationship between cerebrospinal fluid proteins and asthma and to identify potential drug targets. The cerebrospinal fluid proteins IL-6, sRa, and layilin have been identified as potential drug targets. In the PPI network analysis, lililin was found to be associated with three asthma drug targets (IgE, IL-5, and IL-4), further indicating that lililin may be a potential therapeutic target for asthma.

IL1-R1 is a cytokine receptor that serves as the receptor for IL-1α, IL-1β, and IL-1RA. When bound to IL-1α and IL-1β, it activates intracellular signalling pathways [42]. A previous study indicated that during periods of psychological stress in patients with asthma, there is increased glucose metabolism in the amygdala, which is associated with increased IL-1 signalling in the airways, suggesting the existence of a brain immune pathway in asthma [43]. IL1-R1 exhibited a strong interaction with IL-33 in the PPI analysis. Studies have shown that IL-33 encodes a cytokine released during cellular damage, whereas IL1-RL1 encodes a part of the IL-33 receptor complex [44]. Recent advances in functional studies in human participants and mouse models of allergic airway disease suggest that IL-33 signalling plays a central role in driving TH2 inflammation, which is the core of eosinophilic allergic asthma [45]. Based on pharmacogenomic screening, IL-33 is a potential small-molecule therapeutic target. Currently, data from two Phase II clinical trials have shown that targeting IL-33 monoclonal antibodies or IL-33R monoclonal antibodies reduces acute asthma attacks compared to placebo [46, 47]. Additionally, IL1-R1-targeting drugs were found in DrugBank, including Anakinra, SD118, OMS-103HP, and Foreskin fibroblasts (neonatal). As the concept of drug repositioning has been applied to drugs currently marketed or under development, this method can be used to investigate whether the aforementioned four drugs can also effectively treat asthma [48]. As the safety of these drugs has been established, this approach can enhance the efficiency of drug development, while reducing costs and time. IL-7R is also a cytokine receptor that binds to IL-7 or thymic stromal lymphopoietin (TSLP), activates JAK-STAT and other pathways and regulate type 2 inflammation [49]. In ovalbumin-induced allergic asthma mouse models, IL-7 signaling has been shown to be necessary for the survival of allergen-specific CD4 + T cells [50]. Additionally, IL1-R1 was found to interact with IL-7R in the PPI analysis. Currently, there are no studies on combined therapy targeting IL1-R1 and IL-7R, providing new insights for our research on targeted asthma medications.

ECM1 was initially identified as an 85 kDa glycoprotein secreted by the mouse osteoblastic cell line MN7. The human homologue regulates endochondral bone formation, stimulates endothelial cell proliferation, and induces angiogenesis [51]. Li et al. confirmed that ECM1 was elevated and specifically expressed in Th2 cells, leading to exacerbated allergic airway inflammation [52]. Another study found that ECM1 inhibits the differentiation of Th17 cells in inflammatory diseases of the central nervous system; however, inhibiting Th17 cell differentiation can reduce the occurrence of asthma [53]. CD200R1 is an immunoregulatory receptor on the surface of myeloid cells. Upon binding to the cell surface glycoprotein CD200, it transmits immune inhibitory signals, resulting in the suppression of mast cell and eosinophil degranulation and modulation of macrophage function [54]. Lauzon-Joset et al. have demonstrated in animal models that CD200R1 activation eliminates airway hyperresponsiveness in experimental asthma [55]. Combined with previous research, our study found that ECM1 and CD200R1 are risk factors for asthma, indicating that we can systematically obtain more experimental data, including GWAS and basic research, to elucidate this further. Additionally, the colocalization analysis of plasma ECM1 and asthma-shared causal variant sites suggests a higher likelihood of it becoming a potential therapeutic target.

Our study has some limitations. First, we tested the effects of proteins from different studies, and inconsistencies in the measurements between different studies may lead to biased results. Additionally, patients with different types of asthma may exhibit different genetic variations. Second, most proteins have only one cis-acting SNP that is significantly associated with the whole genome (*P* < 5 × 10^−8^), lacking trans-acting pQTLs, which limits the application of analysis, including alternative MR algorithms, heterogeneity testing, and pleiotropy testing. However, our investigation of the main discovered SNPs suggested that most SNPs had F-statistics > 10. Furthermore, the effect allele frequencies of the plasma pQTLs retrieved from matched human genome constructs for ADAM19 were close to 0.5, indicating low reliability in the direction of its effect. Therefore, the effects of ADAM19 should be interpreted with caution. Third, our analysis was conducted on populations of European ancestry, making it difficult to generalise the results to other races. Further research in non-European populations is required to translate these findings to clinical applications. Finally, although we found some interactions between the pathogenic proteins of current asthma medications and drug targets, the results of the PPI analysis were suggestive rather than conclusive, and more research, such as studies using cell lines, animal models, and clinical samples, is needed to validate these findings.

## Conclusion

Our study demonstrated a causal relationship between genetic determinants, including IL1R1, IL7R, ECM1, CD200R1, ADAM19, IL-6 sRa, and Layilin (LAYN) protein levels, and asthma. Additionally, the identified proteins may serve as attractive drug targets for asthma, particularly ECM1 and Layilin (LAYN). However, further research is required to fully understand the roles of these proteins in the onset and progression of asthma. Our findings provide important insights into the discovery of novel therapeutic targets for asthma. Through the integration of Mendelian randomization, drug prediction, phenotype scanning, gene colocalization analysis, protein-protein interaction network construction, and external validation, our study offers valuable guidance for developing more effective and targeted treatment approaches.

## Supplementary Information

Supplementary Material 1: **Supplementary tables. Table S1.**Genetic instruments of plasma and brain proteins for MR analysis. **Table S2.** Genetic instruments of asthma for bidirectional MR.**Table S3.** Heterogeneity analysis on proteins with two or more instruments.**Table S4.** Previously-reported genome-wide significant association of SNPs as genetic instruments of seven potential causal proteins.**Table S5.** Medications for asthma and their corresponding drug targets.**Table S6.** Current medications targeting seven potential causal proteins and interacted proteins.**Table S7.** Colocalization analysis of seven potential causal proteins and asthma.**Table S8.** Genetic instruments of seven potential causal proteins for external validation.

Supplementary Material 2: **: Supplementary figures. Figure S1.** Bidirectional MR analysis for asthma on levels of seven potential causal proteins.**Figure S2.** Bayesian colocalization analysis of seven potential causal proteins and asthma.**Figure S3.** Comparison analysis of MR estimates between plasma proteome and CSF proteome. **Figure S4.** Potential drug target protein-protein interaction network among the suggestive causal proteins (*P* < 0.05). **Figure S5.** Seven identified protein-protein interaction network among the suggestive causal proteins (*P* < 0.05).**Figure S6.** Four asthma drug targets protein-protein interaction network among the suggestive causal proteins (*P* < 0.05).

## Data Availability

No datasets were generated or analysed during the current study.

## Abbreviations

CSF: Cerebrospinal Fluid
GWAS: Genome-Wide Association Studies
MR: Mendelian Randomization
SNP: Single Nucleotide Polymorphism
pQTL: Protein Quantitative Trait Loci
OR: Odds Ratio
IVW: Inverse Variance Weighted
WM: Weighted Mode
WME: Weighted Median Method
SM: Simple Mode
PPI: Protein-Protein Interaction
IL1: R1-Interleukin 1 Receptor Type 1
IL: 7R-Interleukin 7 Receptor
ECM1: Extracellular Matrix Protein 1
CD200R1: CD200 Receptor 1
ADAM19: ADAM Metallopeptidase Domain 19
IL: 6 sRa-Interleukin 6 soluble Receptor alpha
LAYN: Layilin
