# Supplementary material for: Potential drug targets for asthma identified through mendelian randomization analysis
Source: Respir Res. 2025 Jan 13;26:16. doi: 10.1186/s12931-024-03086-5 (PMC11730137; doi:10.1186/s12931-024-03086-5)
Supplement: Supplementary file 2 — Supplementary Material 2: : Supplementary figures. Figure S1. Bidirectional MR analysis for asthma on levels of seven potential causal proteins.Figure S2. Bayesian colocalization analysis of seven potential causal proteins and asthma.Figure S3. Comparison analysis of MR estimates between plasma proteome and CSF proteome. Figure S4. Potential drug target protein-protein interaction network among the suggestive causal proteins (P < 0.05). Figure S5. Seven identified protein-protein interaction network among the suggestive causal proteins (P < 0.05).Figure S6. Four asthma drug targets protein-protein interaction network among the suggestive causal proteins (P < 0.05). [file 12931_2024_3086_MOESM2_ESM.docx]

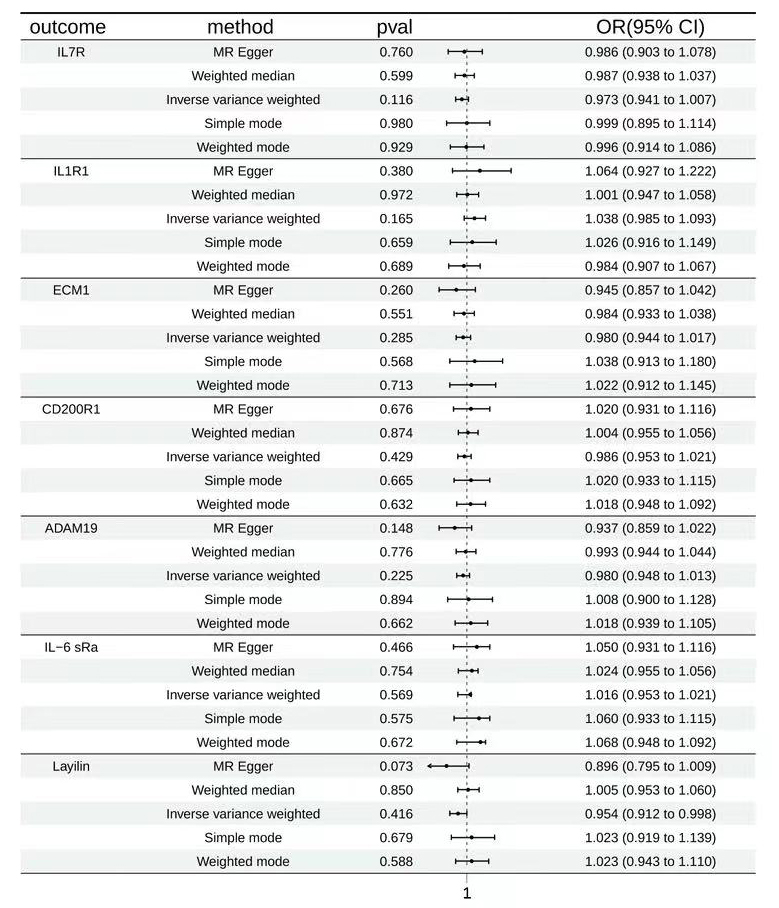


Supplementary Figure S1 Bidirectional MR analysis for asthma on levels of seven potential causal proteins

OR stood for the odds ratios for per standard deviation (SD) increase in plasma protein levels and per 10-fold increase in CSF protein levels as asthma risk increased.


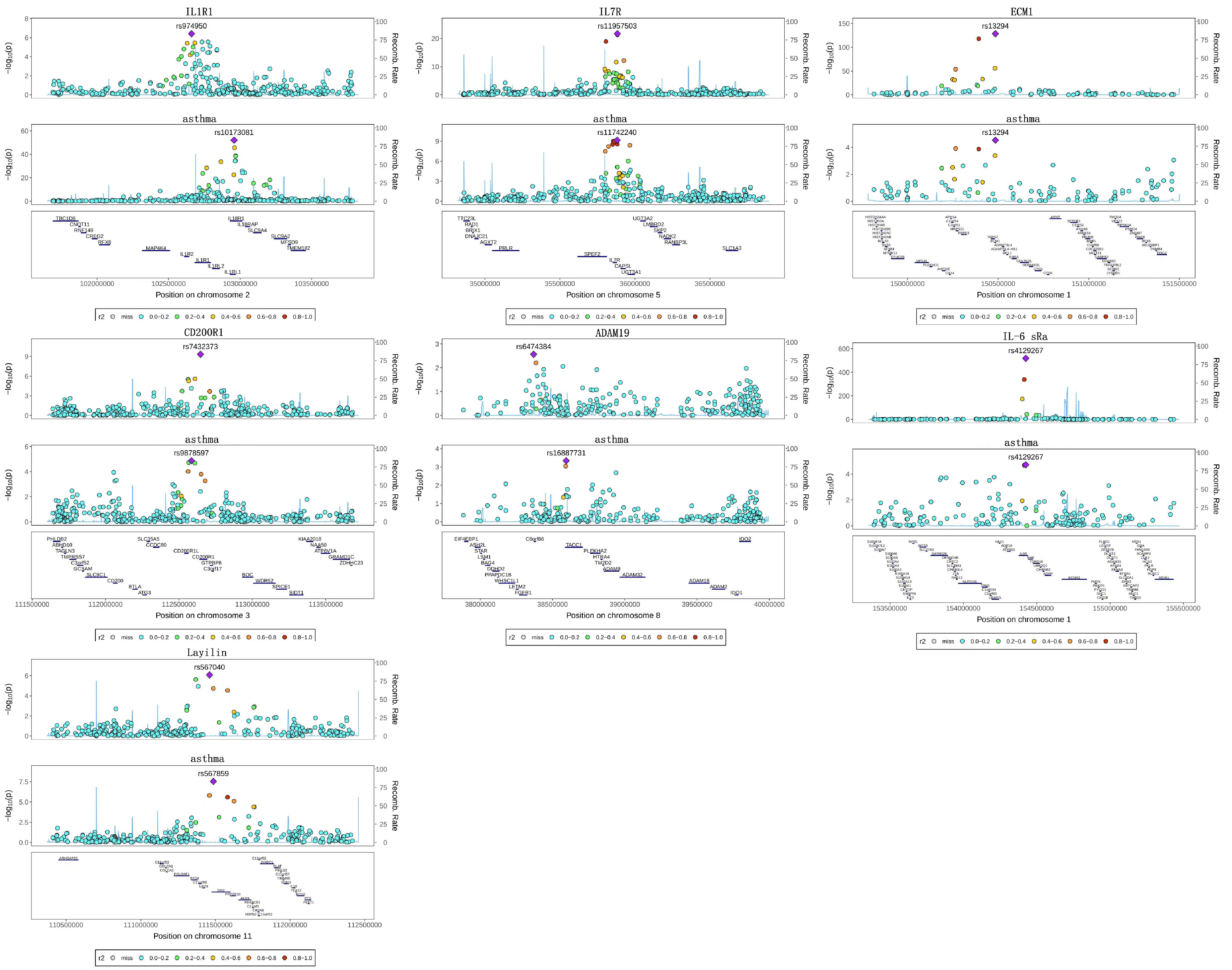


Supplementary Figure S2 Bayesian colocalization analysis of seven potential causal proteins and asthma

Colocalization analysis of plasma proteins and CSF proteins, respectively.

Diamond purple points represented the SNP that with the minimal sum of Pvalue in corresponded protein GWAS and asthma GWAS.


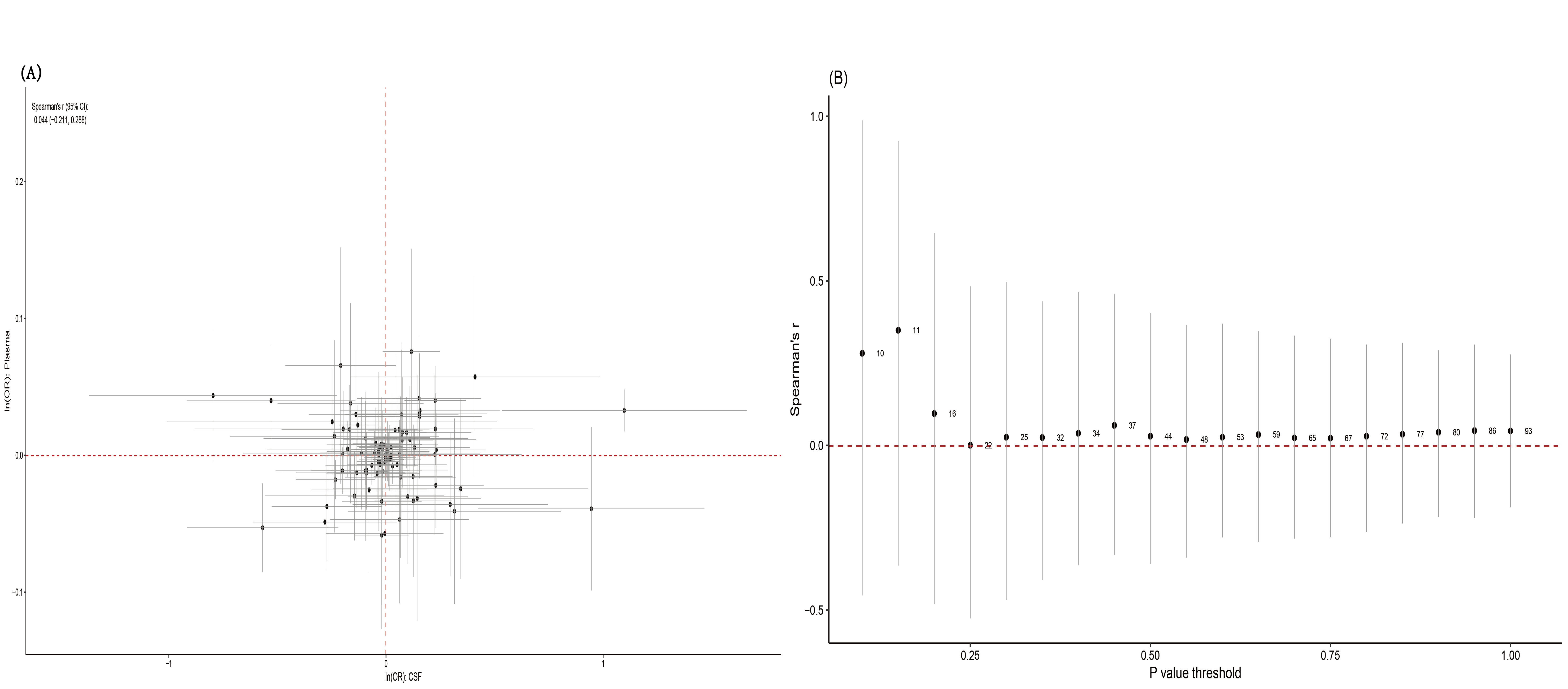


Supplementary Figure S3 Comparison analysis of MR estimates between plasma proteome and CSF proteome

(A)All 66 overlapping proteins in plasmaand CSF were used to perform correlation

analysis. The horizontal and vertical gray line represented the 95% confidence interval

of MR estimates in main analysis. The Spearman correlation coefficient was 0.044 (95% CI: -0.211, 0.288); (B)With different cutoff for Pvalue to include MR estimates,

Spearman correlation coefficient was calculated. The numbers on the left side of the

black point represented the numbers of overlapping proteins correspondingly.


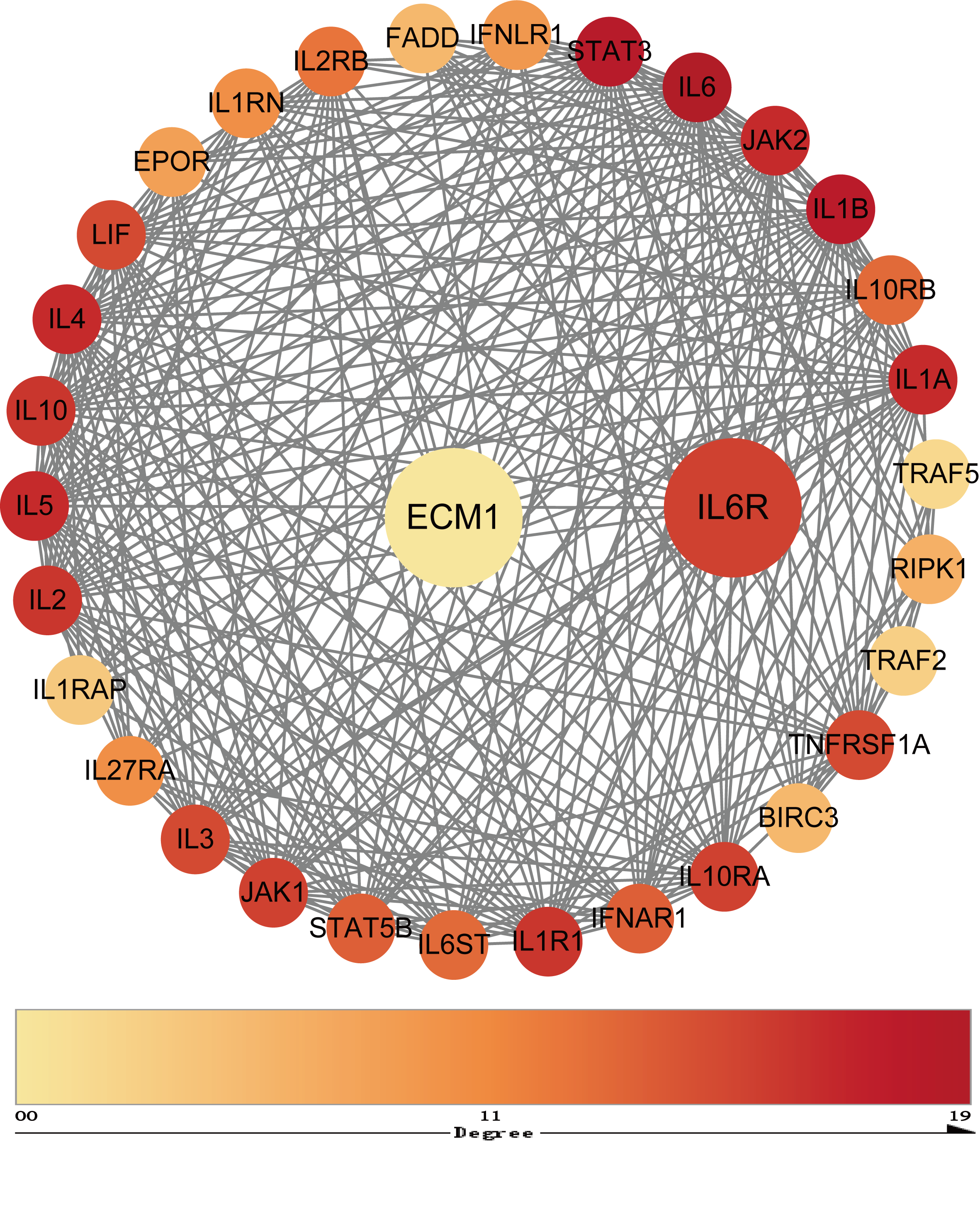


Supplementary Figure S4 Potential drug target protein-protein interaction network among the suggestive causal proteins (P < 0.05)

Different red Degrees represent proteins with different degree levels, and the deeper the red degree, the closer the relationship between proteins.


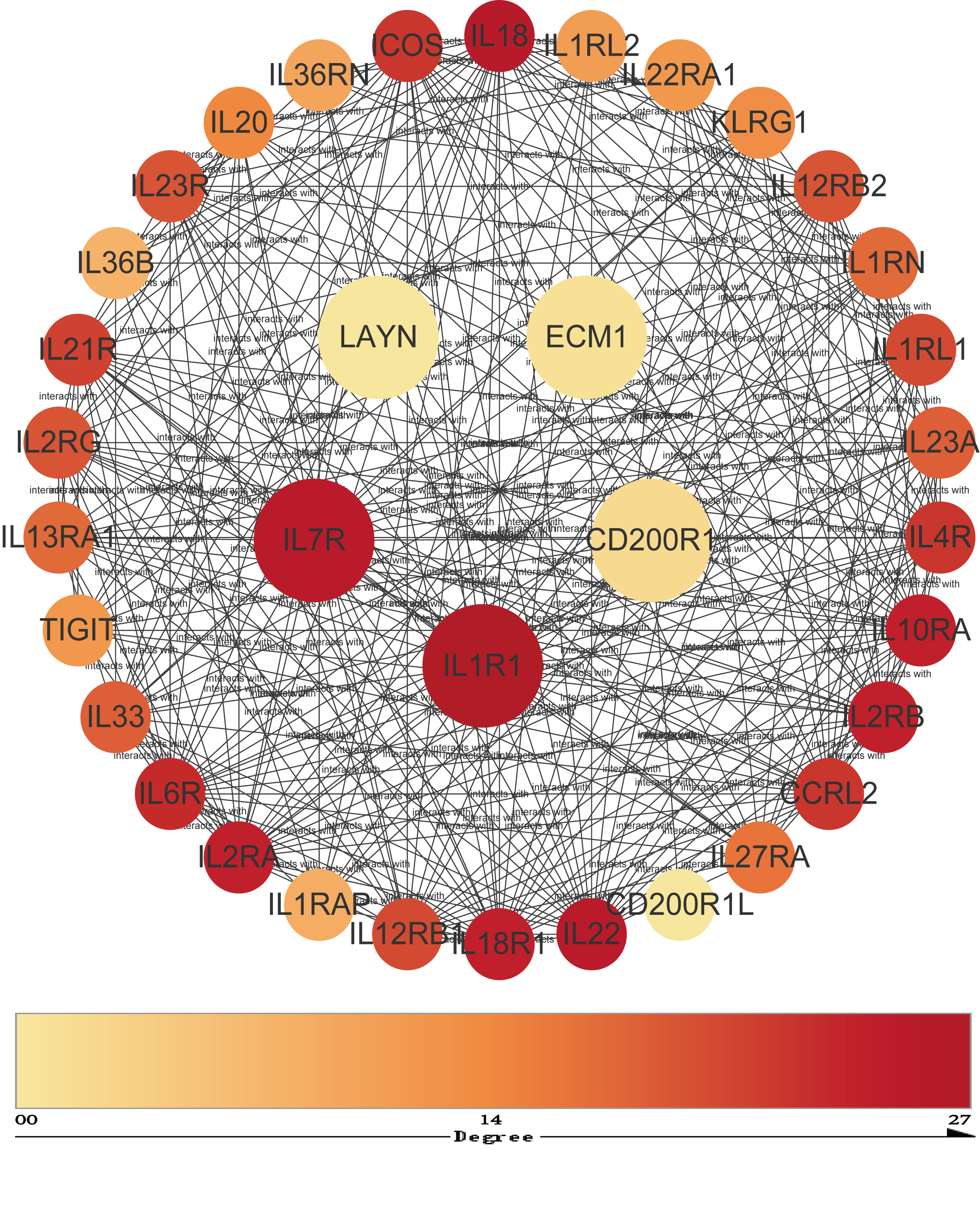


Supplementary Figure S5 Seven identified protein-protein interaction network among the suggestive causal proteins (P < 0.05)

Different red Degrees represent proteins with different degree levels, and the deeper the red degree, the closer the relationship between proteins.


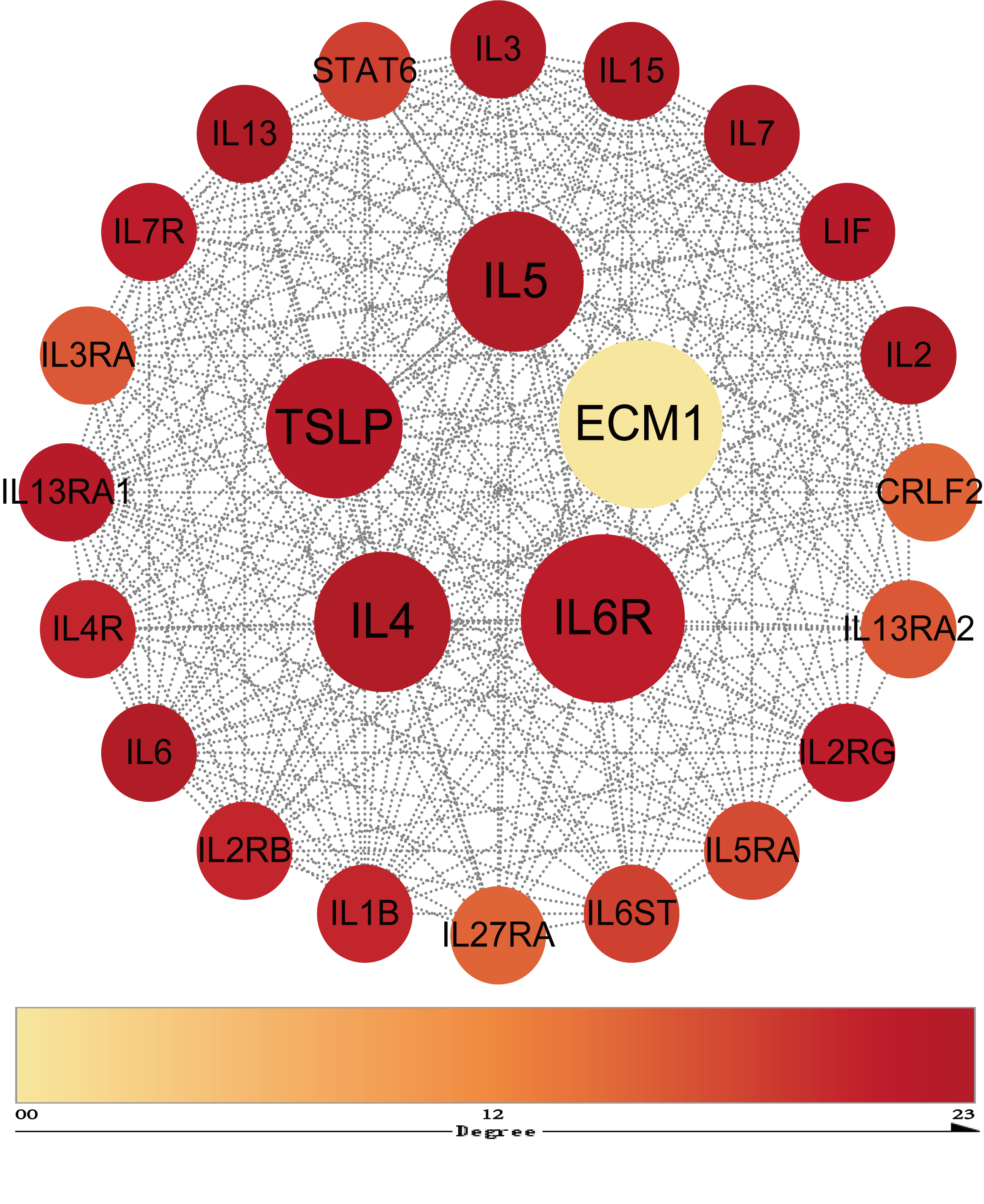


Supplementary Figure S6 Four asthma drug targets protein-protein interaction network among the suggestive causal proteins (P < 0.05)

Different red Degrees represent proteins with different degree levels, and the deeper the red degree, the closer the relationship between proteins.
